# Mesmerism

**Published:** 1886-06

**Authors:** 


### HALL'S JOURNAL OF HEALTH

Vol. 33. JUNE, 1886. No. 6.

### MESMERISM.

When, in 1776, Mesmer, the celebrated German physician, published his treatise entitled "*De Planetarum Influxu*," in which he claimed that the whole universe is pervaded by an element more subtle than the air, which permeates all bodies, and to whose influence the nervous system of all animals responds ; not only those of the medical profession generally, but nearly all who laid claim to a superior knowledge of natural law, rewarded his discoveries by assigning him a place among the charlatans and "cranks" of his time. And when, at a later day, after repeated experiments which confirmed his discovery of facts, the causes of which were yet unexplained and quite inexplicable even to his own mind, upon any known hypothesis, Mesmer published his "*Memoire sur la Decouverte du Magnetisme Animal*," in which he admitted that no reasoning could overcome the difficulties in the way of an acceptance of his discoveries—nothing less, indeed, than *experience*—he was met with indignities serious enough to cause him to quit Paris, where he was then located, for England where he sought protection from his assailants under a feigned name, and eventually died in obscurity in the place of his nativity.

It was then, as it is now, a most unwelcome thing to lead the mind beyond the level of the commonplace in either religion or science, into new and unexplored fields, and more than all, to advance any new theory dependent upon correlative facts, however well established, based upon immateriality, or anything which could not be subjected to physical proof.

This was Mesmer's misfortune. He was able to demonstrate his *facts*, but he could not prove his *theories*, nor could *they* be investigated by any known rule of comparison or analysis, and so, like many another, his life and usefulness were sacrificed to skepticism and ignorance.

Who now that does not believe in mesmerism, and yet how few there are who have any conception of the subtle elements in nature to which this force is assignable.

It is within the recollection of our readers that a leading physician of New York City, and a votary of science, published a work in which he denied the existence of any faculty present in man not solely dependent upon his physical being, and subsequently, when the truth of mesmerism was brought home to him by indisputable evidence he practiced it as an operator before the local medical society of which he was a member, with subjects of his own selection and all the *eclat* of a new discovery, thus demonstrating mesmerism to be not only true in science, but capable of being employed in medical and surgical practice as a valuable auxiliary, as indeed it has been in numberless instances, ranging from the time when to this end, Mesmer employed its mysterious agency in his practice of the healing art more than a century ago, down to the present day.

As early as the year 1776 Mesmer memorialized the Parisian Academy of Sciences, the Academy of Berlin, and the Royal Society of London, calling attention to his discoveries. Only the last named vouchsafed a reply, in which he was regarded as a visionary. In the language of a biographer, he was disregarded by the learned and treated as a juggling impostor by his professional brethren. It has taken a whole century of time to induce the medical profession to acknowledge even partially, the truth and value to science of Mesmer's discoveries

Some recent writers of this class, seemingly reluctant to fittingly honor his name, or to ascribe to unfamiliar causes the phenomenon of mesmerism, have treated of it under the cognomen of *hypnotism*, as if the mesmeric state were a *sleep*, capable of being induced by the administration of drugs. rather than the subjection of the intellectual faculties to the will of another through the exertion of natural forces, which thus far no one has been able to fully comprehend, much less satisfactorily explain. That they relate to the spiritual rather than the physical side of nature, the evidences all tend to show, yet medical science, as taught in the schools, has for the most part habitually opposed this view of the case, and failed to submit a better one, often demonstrating both in theory and practice that "a little learning is a dangerous thing"

The pupils and successors of Messmer do not seem to have availed themselves of the advantages resulting from his discovery to any considerable extent. Indeed, in their day, it was scarcely prudent for them to offer themselves as a mark for the "slings and arrows" of offended pride or wanton ignorance. But in this country some very eminent members of the medical profession have had the independence and temerity, notably during the past thirty years, to investigate, practice and publicly demonstrate the science of mesmerism to be true by a series of startling experiments before large and interested audiences. Among the most noted of these were Doctors Quimby of Portland, and S. B. Brittan of New York, now deceased.

Numerous experiments of late years have been made in demonstrating the existence of the faculty which is called the second sight (double vue), and the perception of persons and objects more or less distant, and through bodies which refract light, or which are quite opaque.

A Viennese professor has founded an institution for the treatment of nervous disorders, whose subjects are especially proper for experimenting in magnetism. This professor at his clinical class one evening drew from his pocket a letter addressed to him by an English correspondent residing in London. This letter, which he had not yet opened, he placed in the hand of one of his subjects, after putting her into the magnetic sleep, and asked her to tell him the contents of the letter contained in the unopened envelope. She saw the writing, but it was English, which she did not understand. The professor said, 'I will that you know English.' The subject then read the letter in English, as well as an English person might do. 'Now,' said he, 'translate into German.' The translation was exact and lucid. The subject of the letter was upon a certain point in physiology, and it abounded in technical terms. He then told the subject to describe the writer of the letter. She did so, saying that he was seated at a table in a study, which she also described. The professor wrote to his English correspondent, asking him to send him his photograph, and also a photograph of his study; and the description by the magnetised subject was found to be exact. Experiments with this faculty of the subject have been made ten times without failure.

The writer has been an observer of many curious experiments with mesmeric sensitives under the direction of well-known mesmerists and lecturers. Without being able to go into scientific details he is fortified at all points by incontrovertable facts which prove the existence of the mesmeric state to be as well established as any known fact in natural science, and yet there are to be found even now numbers of the medical profession, which of all others ought to know this truth, ignorant enough to deny it. Had they lived in the days of that renowned practitioner, Doctor Sangrado, they would have been content to continue their little educational round of bleeding and warm water for all manner of ailments.

